# Sex Difference Trend in 5-Year Mortality Among Patients With Coronary Artery Disease: A 24,432 Chinese Cohort Study From 2007 to 2014

**DOI:** 10.3389/fcvm.2022.774365

**Published:** 2022-04-12

**Authors:** Haozhang Huang, Wenguang Lai, Qiang Li, Haiyan Wei, Nuerbahaer Remutula, Tilakezi Tuersun, Zhou Yang, Kunming Bao, Zelin Yan, Bo Wang, Yibo He, Shiqun Chen, Chun-Quan Ou, Heyin Yang, Jiyan Chen, Jin Liu, Yong Liu

**Affiliations:** ^1^Department of Cardiology, Guangdong Cardiovascular Institute, Guangdong Provincial People's Hospital, Guangdong Academy of Medical Sciences, Guangzhou, China; ^2^Department of Guangdong Provincial Key Laboratory of Coronary Heart Disease Prevention, Guangdong Cardiovascular Institute, Guangdong Provincial People's Hospital, Guangdong Academy of Medical Sciences, Guangzhou, China; ^3^The Second School of Clinical Medicine, Southern Medical University, Guangzhou, China; ^4^Guangdong Provincial People's Hospital, School of Medicine, South China University of Technology, Guangzhou, China; ^5^The First People's Hospital of Kashgar Prefecture, Kashgar, China; ^6^State Key Laboratory of Organ Failure Research, Department of Biostatistics, Guangdong Provincial Key Laboratory of Tropical Disease Research, School of Public Health, Southern Medical University, Guangzhou, China; ^7^Department of Cardiology, Longyan First Affiliated Hospital of Fujian Medical University, Longyan, China

**Keywords:** gender, sex differences, coronary artery disease, mortality, trend

## Abstract

**Background:**

The sex difference trend of short-term mortality in coronary artery disease (CAD) is narrowing, which has been reported in the previous studies. However, no studies assess the sex difference temporal trends of CAD mortality in China especially long-term mortality trend.

**Methods:**

Based on the registry at Guangdong Provincial People's Hospital which is the largest cardiovascular center in South China, this retrospective cohort study included 24,432 hospitalized patients with CAD confirmed by coronary angiography from January 2007 to December 2014. Women and men were followed for 1-year and 5-year all-cause mortality.

**Results:**

From 2007 to 2014, 5-year age-standardized mortality increased from 10.0 to 11.7% in men (*p* for trend < 0.001) and from 11.5 to 8.1% in women (*p* for trend = 0.99). The multivariable-adjusted hazard ratios (95% CI), which compare women with men, were from 1.02 (0.39–2.67) to 0.66 (0.39–1.12) for 1-year all-cause mortality and 1.23 (0.64–2.36) to 0.59 (0.44–0.79) for 5-year all-cause mortality (*p* for trend = 0.04).

**Conclusion:**

Our study found that the mortality risk among men and women was similar in the 1-year prognosis of CAD, and there was no significant downward trend. In the 5-year long-term prognosis of CAD, the mortality risk among men continued to rise, while women had reached the peak, which means that the mortality risk continues to be higher among men than women.

## Introduction

Global Burden of Disease study indicated that coronary artery disease (CAD) remains the leading cause of morbidity and mortality (1). Over the past decade, scientists, healthcare providers, the public, and policymakers have made substantial efforts to improve the understanding of the gender differences among patients with CAD.

It is well-known that CAD mortality is higher among men than women in the previous studies (2–6). While several studies indicate that women have experienced frustratingly smaller decreases compared with men in CAD mortality rates (6, 7). In addition, there is an observed narrowing of sex inequalities in CAD mortality in keeping with a previous WHO report (8, 9).

In China, the largest developing country with the highest burden of ischemic heart disease (1), the social protection and the economic system are different from those of European and American countries. Sex difference temporal trends of mortality are still unknown among hospitalized patients with CAD in China especially long-term mortality trends.

Therefore, based on the 11 years data of a sample of 24,432 people in the largest cardiovascular disease center in South China, we aim at assessing the sex difference temporal trends of short-term and long-term mortality after CAD admission in China.

## Method

### Data Sources and Study Population

This retrospective observational study was based on the registry of Cardiorenal Improvement (CIN) Cohort from January 2007 to December 2014 Guangdong Provincial People's Hospital (GDPH) in China (ClinicalTrials.gov NCT04407936). A total of 51,166 patients underwent coronary angiography (CAG) procedures between 2007 and 2014, and 34,576 patients were diagnosed as CAD; We excluded < 18 years old (*n* = 8), prior myocardial infarction, prior underwent percutaneous coronary intervention (PCI) or coronary artery bypass grafting (*n* = 4,729), and missing follow-up information (*n* = 5,407), which included 24,432 patients with a final diagnosis of CAD (aged at least 18 years old) who are the first-time undergoing CAG (Supplementary Figure 1).

From January 2007 to December 2014, data were extracted from the GDPH electronic clinical management system. The key variables in care for patients with CAD included demographic characteristics (e.g., age, sex, and insurance categories), medical comorbidities, laboratory examinations, procedural types, and medications at discharge. CAG or PCI was performed in accordance with the standard clinical practice guidelines (10–12). During this period of time, only the information of the first hospitalization was taken.

Senior cardiologists were responsible for the data quality control and periodically underwent database checking. Finally, the accuracy of diagnosis and variables based on the medical records have been undergoing random on-site audits. We randomly checked the accuracy of data from the Cardiorenal Improvement (CIN) dataset for 1,500 patients, and the error rate was 0.3% for diagnosis and 0.1% for procedural information. During the study period, the information on the date of death was based on the Guangdong Public Security System, which linked to CIN dataset by the unique identified number.

The study protocol was approved by the GDPH ethics committee (no. GDREC 2019555H[R1]), and the study was performed according to the Declaration of Helsinki. All traceable personal identifiers were removed from the analytic dataset to protect patients' privacy, and the requirement for informed consent was therefore waived.

### Clinical Definition

Coronary artery disease was confirmed by CAG and discriminates it according to the 10th Revision Codes of the International Classification of Diseases. The estimated glomerular filtration rate (eGFR) was calculated by the Modification of Diet in Renal Disease (MDRD) formula. Comorbidities include hypertension, DM, congestive heart failure (CHF, was defined as New York Heart Association (NYHA) class > 2 or Killip class > 1), atrial fibrillation or flutter (AF), chronic kidney disease (CKD, was defined as eGFR ≤ 60 ml/min/1.73 m^2^), (13) chronic obstructive pulmonary disease (COPD), and tumors; medications include aspirin, clopidogrel, angiotensin-converting enzyme inhibitors or angiotensin receptor blockers (ACEI/ARB), beta-blockers, statins, and calcium channel blockers (CCB).

### Study Outcome

The 1-year and 5-year mortality after admission were the primary outcomes. The overall proportion of age and sex structure, comorbidities, and medications were described. Finally, we present the annual trend in age-standardized mortality from 2007 to 2014 among women and men.

### Statistical Analysis

We described the demographics, discharge diagnosis, treatment (procedure), examination, medication use, and discharge status of patients with CAD, which were computed by gender and time. Except for the examination and contrast medium volume and examination variables, the continuous variables were used mean (standard deviation [SD]) to describe. Other characteristics were categorical variables, and they were presented as counts and proportions. Differences between each calendar year were examined with one-way analysis of variance (ANOVA) for continuous variables and Pearson's chi-squared tests for dichotomous variables, using Fisher's exact test when needed (refer to Supplementary Table 1).

To assess the annual changes of these characteristics among patients with CAD stratified by gender from 2007 to 2014, linear regression model was fitted on each continuous variable, whereas a logistic regression model was fitted on each categorical variable. In these models, the independent variable was the calendar year as a numerical variable. By testing the statistical significance of the regression coefficient of the calendar year variable, we can determine the presence of the trend (**Tables 2**, **3**).

To evaluate the temporal trends of patients with CAD within 1 year of admission and within 5 years of admission, we calculated the sex- and age-adjusted standardized mortality rates each year. Patients aged 20 to 90 were divided into 14 groups for every 5 years of age, whereas the patients younger than 20 or older than 90 were allocated into two groups, respectively. Each age-standardized mortality rate was calculated by adding up the total number of patients in each age group from 2007 to 2014 as the standard population. We then evaluated the sex-specified and age-adjusted mortality rates to compare the demographic composition and mortality of male and female patients with CAD.

We also used Cox regression to estimate the female-to-male hazard ratio (HR), and 95% confidence interval (CI) for each of the annual trends was analyzed by using Cox models, in which the calendar year was taken as the numerical independent variable. The following model was sequentially constructed with or without adjustment for covariates: demographic characteristics (age and insurance type), diagnosis (hypertension, CKDs, acute myocardial infarction, congestive heart failure, stroke, diabetes mellitus, percutaneous coronary intervention, AF COPD, cancer, and anemia). As for the selection of the above covariates, we referred to the previous studies and took into account the clinical factors that may have an influence on the mortality of patients with CAD.

### Subgroup Analysis

In addition, we examined the age-standardized mortality trends in different subgroups stratified by age group (<60, 60–74, ≥75 years old). All *p*-values were two-sided, and *p*-values <0.05 were considered significant. All statistical analyses were performed using R (version 4.0.3).

## Result

### Clinical Characteristics

**Figure 1:**
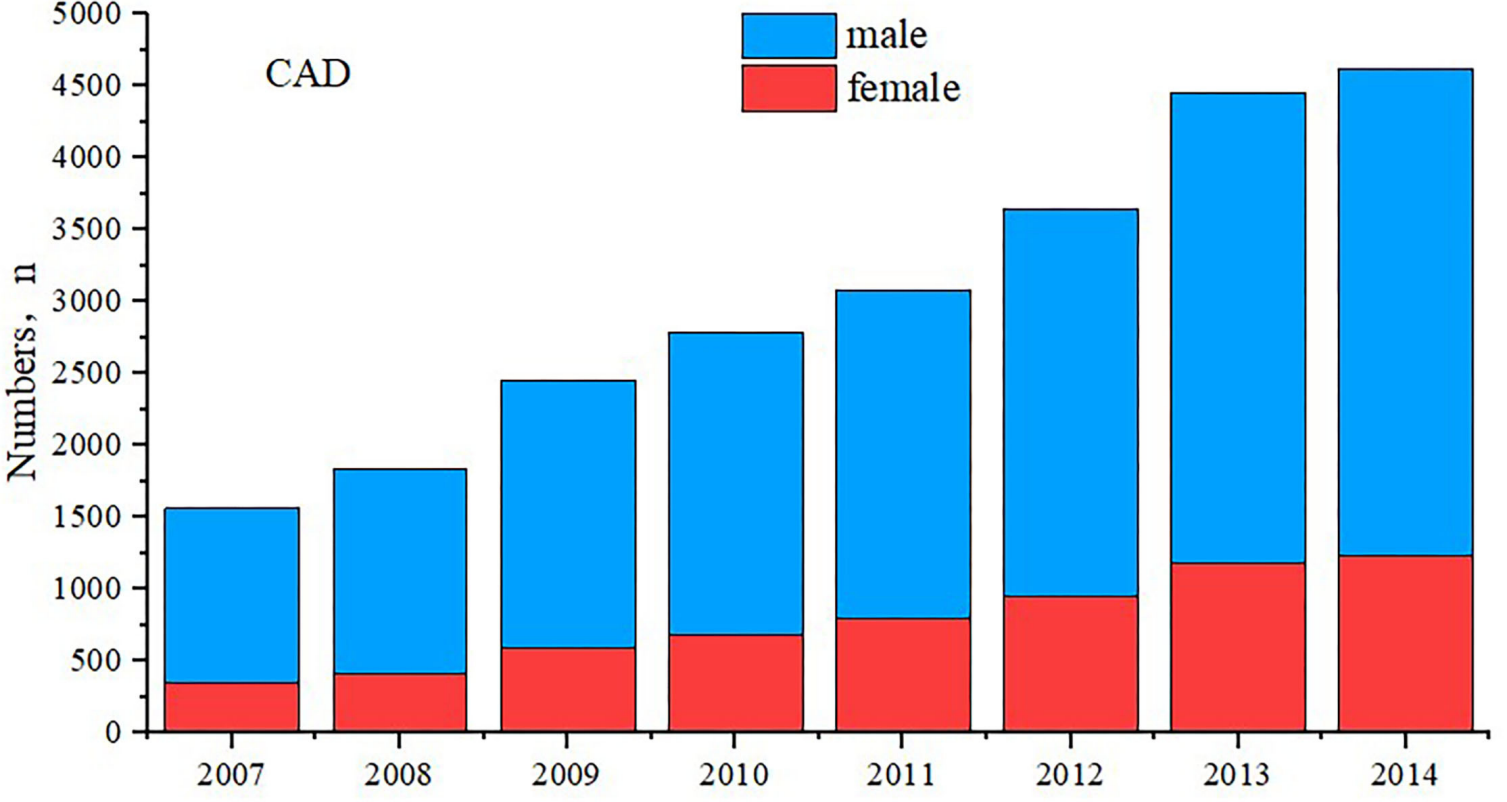
Patients admitted between January 2007 and December 2017.

A total of 24,432 patients with CAD from January 2007 to December 2014 were included in this study (Tables 1–3 and Supplementary Table 1). The number of CAD increased gradually, in both men and women (Figure 1). For the demonstration purposes, detailed baseline characteristics of the study cohort across gender (male and female) are shown in Table 1. Among these patients, the mean age was 63.0 ± 10.7 years, and 18,224 (74.6%) were men. There were 18,006 (73.7%) patients who underwent PCI and 5,344 (21.9%) patients diagnosed as AMI. Women were negatively associated with AMI, PCI, and the use of the related drug and were positively associated with DM, hypertension, anemia, and CKD morbidity. More details of the baseline characteristics of patients enrolled are shown in Table 1.

**Table 1:** Clinical and biochemical characteristics of patients with CAD stratified by male and female.

**Characteristic**	**Overall (*N* = 24,432)**	**Male (*N* = 18,224)**	**Female (*N* = 6,208)**	***p*-value**
**Demographics**, ***n*** **(%)**				
Age, (years), mean (SD)	62.96 (10.72)	61.84 (10.86)	66.25 (9.55)	<0.001
<60	9,020 (36.9)	7,542 (41.4)	1,478 (23.8)	<0.001
60–75	11,716 (48.0)	8,300 (45.5)	3,416 (55.0)	
≥75	3,696 (15.1)	2,382 (13.1)	1,314 (21.2)	
**Insurance type**, ***n*** **(%)**				
Self-paying	5,436 (22.3)	4,240 (23.3)	1,196 (19.3)	<0.001
Urban insurance	13,168 (54.0)	9,718 (53.5)	3,450 (55.7)	
Rural insurance	1,407 (5.8)	1,098 (6.0)	309 (5.0)	
Other	4,358 (17.9)	3,122 (17.2)	1,236 (20.0)	
**Discharge diagnosis**, ***n*** **(%)**				
AMI	5,344 (21.9)	4,446 (24.4)	898 (14.5)	<0.001
HT	13,616 (55.7)	9,470 (52.0)	4,146 (66.8)	<0.001
DM	6,292 (25.8)	4,310 (23.7)	1,982 (31.9)	<0.001
AF	701 (2.9)	474 (2.6)	227 (3.7)	<0.001
CHF	1,772 (15.6)	1,317 (16.0)	455 (14.6)	0.062
Stroke	1,162 (4.8)	855 (4.7)	307 (4.9)	0.438
Cancer	268 (1.1)	184 (1.0)	84 (1.4)	0.030
CKD	5,179 (22.5)	3,651 (21.3)	1,528 (25.9)	<0.001
COPD				
**Procedure**, ***n*** **(%)**				
PCI	18,006 (73.7)	13,892 (76.2)	4,114 (66.3)	<0.001
DES	16,857 (69.0)	12,989 (71.3)	3,868 (62.3)	<0.001
BES	1,114 (4.6)	881 (4.8)	233 (3.8)	<0.001
CMV, mean (SD)	144.49 (82.40)	149.44 (84.02)	130.09 (75.66)	<0.001
**Biochemical characteristics**				
eGFR, mean (SD)	78.28 (25.05)	78.74 (24.22)	76.94 (27.28)	<0.001
HGB, mean (SD)	133.06 (16.73)	136.37 (16.17)	123.44 (14.48)	<0.001
LDL-C, median [IQR]	2.65 [2.09, 3.31]	2.64 [2.08, 3.28]	2.71 [2.12, 3.38]	<0.001
HDLC, median [IQR]	0.97 [0.82, 1.15]	0.94 [0.80, 1.11]	1.07 [0.90, 1.26]	<0.001
HbA1c, mean (SD)	6.56 (1.39)	6.50 (1.37)	6.70 (1.43)	<0.001
LVEF, mean (SD)	59.70 (11.85)	58.95 (12.04)	61.92 (10.98)	<0.001
**Medication**, ***n*** **(%)**				
ACEI/ARB	13,194 (55.3)	10,185 (57.2)	3,009 (49.7)	<0.001
β-blocker	19,681 (82.4)	14,783 (83.0)	4,898 (80.9)	<0.001
Statins	22,404 (93.8)	16,865 (94.6)	5,539 (91.5)	<0.001
Aspirin	21,586 (90.4)	16,345 (91.7)	5,241 (86.6)	<0.001
Clopidogrel	20,380 (85.4)	15,648 (87.8)	4,732 (78.2)	<0.001
CCB	5,205 (21.8)	3,526 (19.8)	1,679 (27.7)	<0.001

*ACEI/ARB, angiotensin-converting enzyme inhibitor/angiotensin receptor blocker. CCB, calcium channel blocker; CHF, congestive heart failure; CKD, chronic kidney disease; DM, diabetes mellitus; eGFR, estimated glomerular filtration rate; HbA1c, hemoglobin A1c; LDL-C, low-density lipoprotein cholesterol; LVEF, left ventricular ejection fraction; PCI, percutaneous coronary intervention*.

There was a significant difference in the trend of men and women who were receiving PCI. There was no significant change in the proportion of men who were receiving PCI, but the proportion of women who were receiving PCI was declining (*p* for trend < 0.001). Among male group, the proportion of HT and DM increased year by year (*p* for trend < 0.05) while the proportion of HT and DM did not increase among female group (*p* for trend > 0.05). The proportion of CKD, CHF, and hyperlipidemia increases year by year in both men and women (*p* for trend < 0.05). Additionally, there was no significant difference between men and women with hypertension and diabetes (Tables 2, 3).

**Table 2:** Clinical and biochemical characteristics of male patients with CAD from 2007 to 2014.

**Characteristic**	**Overall (*N* = 18,224)**	**2007** **(*N* = 1,215)**	**2008** **(*N* = 1,419)**	**2009** **(*N* = 1,868)**	**2010** **(*N* = 2,103)**	**2011** **(*N* = 2,284)**	**2012** **(*N* = 2,685)**	**2013** **(*N* = 3,264)**	**2014** **(*N* = 3,386)**	***P* for trend**
**Demographics**, ***n*** **(%)**									
Age, (years), mean (SD)	61.8 (10.9)	61.4 (11.1)	61.1 (10.9)	62.1 (11.0)	61.9 (11.0)	61.7 (10.8)	61.8 (10.9)	62.0 (10.8)	62.2 (10.7)	<0.001
<60	7,542 (41.4)	521 (42.9)	625 (44.0)	747 (40.0)	876 (41.7)	947 (41.5)	1,118 (41.6)	1,336 (40.9)	1,372 (40.5)	0.085
60–75	8,300 (45.5)	562 (46.3)	619 (43.6)	878 (47.0)	947 (45.0)	1,050 (46.0)	1,209 (45.0)	1,475 (45.2)	1,560 (46.1) 0.89	
≥75	2,382 (13.1)	132 (10.9)	175 (12.3)	243 (13.0)	280 (13.3)	287 (12.6)	358 (13.3)	453 (13.9)	454 (13.4)	0.0207
**Insurance Type**, ***n*** **(%)**										
Self-paying	4,240 (23.3)	809 (66.6)	807 (56.9)	481 (25.7)	543 (25.8)	480 (21.0)	305 (11.4)	457 (14.2)	358 (10.6)	<0.001
Urban insurance	9,718 (53.5)	298 (24.5)	450 (31.7)	949 (50.8)	1,049 (49.9)	1,264 (55.3)	1,552 (57.8)	1,909 (59.3)	2,247 (66.4) <0.001	
Rural insurance	1,098 (6.0)	99 (8.1)	114 (8.0)	143 (7.7)	157 (7.5)	133 (5.8)	149 (5.6)	145 (4.5)	158 (4.7)	<0.001
other	3,122 (17.2)	9 (0.7)	48 (3.4)	295 (15.8)	354 (16.8)	407 (17.8)	677 (25.2)	709 (22.0)	623 (18.4)	<0.001
**Discharge Diagnosis**, ***n*** **(%)**									
AMI	4,446 (24.4)	429 (35.3)	426 (30.0)	496 (26.6)	437 (20.8)	442 (19.4)	623 (23.2)	732 (22.4)	861 (25.4)	<0.001
HT	9,470 (52.0)	583 (48.0)	730 (51.4)	955 (51.1)	1,015 (48.3)	1,207 (52.8)	1,399 (52.1)	1,726 (52.9)	1,855 (54.8)	<0.001
DM	4,310 (23.7)	252 (20.7)	301 (21.2)	436 (23.3)	488 (23.2)	580 (25.4)	656 (24.4)	780 (23.9)	817 (24.1)	0.0049
AF	474 (2.6)	33 (2.7)	33 (2.3)	42 (2.2)	54 (2.6)	62 (2.7)	83 (3.1)	74 (2.3)	93 (2.7)	0.577
CHF	1,317 (16.0)	80 (11.2)	79 (10.1)	88 (9.2)	66 (8.3)	61 (5.9)	180 (15.4)	358 (27.1)	405 (28.0)	<0.001
Stroke	855 (4.7)	46 (3.8)	56 (3.9)	85 (4.6)	75 (3.6)	106 (4.6)	121 (4.5)	158 (4.8)	208 (6.1)	<0.001
Cancer	184 (1.0)	11 (0.9)	20 (1.4)	26 (1.4)	24 (1.1)	26 (1.1)	17 (0.6)	27 (0.8)	33 (1.0)	0.317
CKD	3,651 (21.3)	299 (28.1)	300 (24.2)	449 (27.4)	385 (19.6)	411 (18.4)	483 (18.7)	631 (19.9)	693 (21.2)	<0.001
COPD	182 (1.0)	16 (1.3)	12 (0.8)	23 (1.2)	19 (0.9)	14 (0.6)	18 (0.7)	21 (0.6)	59 (1.7)	0.341
**Procedure**, ***n*** **(%)**									
PCI	13,892 (76.2)	873 (71.9)	1,049 (73.9)	1,464 (78.4)	1,624 (77.2)	1,801 (78.9)	2,052 (76.4)	2,457 (75.3)	2,572 (76.0)	0.317
DES	12,989 (71.3)	614 (50.5)	950 (66.9)	1,351 (72.3)	1,487 (70.7)	1,709 (74.8)	1,987 (74.0)	2,388 (73.2)	2,503 (73.9)	<0.001
BES	881 (4.8)	305 (25.1)	109 (7.7)	136 (7.3)	130 (6.2)	77 (3.4)	46 (1.7)	47 (1.4)	31 (0.9)	<0.001
CMV, mean (SD)	149.4 (84.0)	169.4 (97.0)	162.4 (93.5)	164.4 (89.8)	161.1 (91.1)	150.4 (82.5)	141.3 (78.0)	140.9 (80.1)	136.1 (71.5)	<0.001
**Biochemical characteristics**									
eGFR, mean (SD)	78.7 (24.2)	73.3 (20.4)	73.0 (20.5)	71.8 (21.6)	78.9 (23.3)	81.8 (25.7)	83.4 (25.4)	79.9 (24.1)	79.4 (25.0)	<0.001
HGB, mean (SD)	136.4 (16.2)	137.1 (16.7)	138.0 (16.3)	136.0 (16.3)	135.9 (16.0)	136.2 (16.1)	136.0 (15.7)	137.3 (15.8)	135.6 (16.7)	0.047
LDL-C, median [IQR]	2.64 [2.08, 3.28]	2.49 [1.96, 3.07]	2.40 [1.90, 2.92]	2.76 [2.16, 3.35]	2.66 [2.12, 3.28]	2.54 [2.01, 3.14]	2.68 [2.07, 3.36]	2.78 [2.19, 3.48]	2.64 [2.06, 3.34]	<0.001
HDLC, median [IQR]	0.94 [0.80, 1.11]	1.00 [0.85, 1.19]	1.00 [0.85, 1.19]	0.97 [0.81, 1.15]	0.96 [0.80, 1.15]	0.87 [0.74, 1.03]	0.91 [0.78, 1.07]	0.94 [0.81, 1.09]	0.93 [0.80, 1.09]	<0.001
HbA1c, mean (SD)	6.50 (1.37)	6.41 (1.30)	6.53 (1.35)	6.49 (1.22)	6.47 (1.33)	6.60 (1.50)	6.58 (1.34)	6.47 (1.40)	6.43 (1.37)	0.386
LVEF, mean (SD)	59.0 (12.0)	59.2 (12.2)	58.6 (12.4)	59.3 (12.7)	59.6 (11.7)	58.4 (12.1)	59.2 (11.7)	58.8 (12.0)	58.7 (11.8)	0.183
**Medication**, ***n*** **(%)**									
ACEI/ARB	10,185 (57.2)	772 (65.6)	927 (67.0)	1,283 (70.3)	1,262 (61.7)	1,336 (59.9)	1,484 (56.3)	1,535 (48.0)	1,586 (47.7)	<0.001
β-blocker	14,783 (83.0)	969 (82.3)	1,172 (84.7)	1,535 (84.2)	1,699 (83.1)	1,859 (83.3)	2,228 (84.5)	2,649 (82.8)	2,672 (80.4)	0.004
Statins	16,865 (94.6)	1,050 (89.2)	1,255 (90.7)	1,732 (95.0)	1,940 (94.9)	2,135 (95.7)	2,532 (96.0)	3,057 (95.6)	3,164 (95.2)	<0.001
Aspirin	16,345 (91.7)	1,078 (91.6)	1,246 (90.1)	1,686 (92.4)	1,890 (92.4)	2,048 (91.8)	2,430 (92.1)	2,936 (91.8)	3,031 (91.2)	0.943
Clopidogrel	15,648 (87.8)	1,059 (90.0)	1,196 (86.5)	1,623 (89.0)	1,825 (89.2)	2,015 (90.3)	2,323 (88.1)	2,772 (86.7)	2,835 (85.3)	<0.001
CCB	3,526 (19.8)	238 (20.2)	307 (22.2)	432 (23.7)	400 (19.6)	433 (19.4)	473 (17.9)	668 (20.9)	575 (17.3)	<0.001

**Table 3:** Clinical and biochemical characteristics of female patients with CAD from 2007 to 2014.

**Characteristic**	**Overall (*N* = 6,208)**	**2007** **(*N* = 347)**	**2008** **(*N* = 418)**	**2009** **(*N* = 586)**	**2010** **(*N* = 687)**	**2011** **(*N* = 798)**	**2012** **(*N* = 955)**	**2013** **(*N* = 1,184)**	**2014** **(*N* = 1,233)**	***P* for trend**
**Demographics**, ***n*** **(%)**										
Age, (years), mean (SD)	66.3 (9.6)	65.6 (9.2)	66.4 (9.1)	66.8 (9.5)	66.6 (9.7)	66.1 (9.6)	66.0 (9.9)	66.3 (9.5)	66.2 (9.4)	0.758
<60	1,478 (23.8)	83 (23.9)	86 (20.6)	132 (22.5)	155 (22.6)	194 (24.3)	252 (26.4)	281 (23.7)	295 (23.9)	0.209
60–75	3,416 (55.0)	214 (61.7)	260 (62.2)	325 (55.5)	380 (55.3)	432 (54.1)	493 (51.6)	647 (54.6)	665 (53.9)	0.001
≥75	1,314 (21.2)	50 (14.4)	72 (17.2)	129 (22.0)	152 (22.1)	172 (21.6)	210 (22.0)	256 (21.6)	273 (22.1)	0.008
**Insurance type**, ***n*** **(%)**										
Self-paying	1,196 (19.3)	202 (58.2)	212 (50.7)	136 (23.2)	137 (19.9)	144 (18.0)	91 (9.5)	139 (11.9)	135 (10.9)	<0.001
Urban insurance	3,450 (55.7)	116 (33.4)	166 (39.7)	314 (53.6)	395 (57.5)	457 (57.3)	537 (56.3)	685 (58.6)	780 (63.3) <0.001	
Rural insurance	309 (5.0)	25 (7.2)	24 (5.7)	45 (7.7)	27 (3.9)	39 (4.9)	47 (4.9)	55 (4.7)	47 (3.8)	0.002
other	1,236 (20.0)	4 (1.2)	16 (3.8)	91 (15.5)	128 (18.6)	158 (19.8)	279 (29.2)	289 (24.7)	271 (22.0) <0.001	
**Discharge Diagnosis**, ***n*** **(%)**										
AMI	898 (14.5)	63 (18.2)	85 (20.3)	93 (15.9)	81 (11.8)	86 (10.8)	151 (15.8)	159 (13.4)	180 (14.6)	0.028
HT	4,146 (66.8)	224 (64.6)	286 (68.4)	392 (66.9)	444 (64.6)	539 (67.5)	638 (66.8)	775 (65.5)	848 (68.8)	0.401
DM	1,982 (31.9)	94 (27.1)	128 (30.6)	191 (32.6)	218 (31.7)	264 (33.1)	315 (33.0)	366 (30.9)	406 (32.9)	0.20
AF	227 (3.7)	16 (4.6)	17 (4.1)	20 (3.4)	23 (3.3)	32 (4.0)	32 (3.4)	45 (3.8)	42 (3.4)	0.418
CHF	455 (14.6)	27 (12.4)	26 (9.6)	28 (8.1)	16 (5.1)	18 (4.6)	70 (15.2)	148 (26.1)	122 (22.0)	<0.001
Stroke	307 (4.9)	9 (2.6)	22 (5.3)	24 (4.1)	32 (4.7)	39 (4.9)	52 (5.4)	62 (5.2)	67 (5.4)	0.053
Cancer	84 (1.4)	2 (0.6)	6 (1.4)	12 (2.0)	7 (1.0)	14 (1.8)	18 (1.9)	11 (0.9)	14 (1.1)	0.638
CKD	1,528 (25.9)	100 (31.9)	133 (35.2)	175 (32.9)	169 (26.2)	185 (23.8)	217 (23.5)	274 (23.9)	275 (23.1)	<0.001
COPD	31 (0.5)	2 (0.6)	2 (0.5)	1 (0.2)	3 (0.4)	2 (0.3)	6 (0.6)	7 (0.6)	8 (0.6)	0.296
**Procedure**, ***n*** **(%)**										
PCI	4,114 (66.3)	232 (66.9)	295 (70.6)	407 (69.5)	480 (69.9)	526 (65.9)	619 (64.8)	729 (61.6)	826 (67.0)	<0.001
DES	3,868 (62.3)	163 (47.0)	268 (64.1)	359 (61.3)	444 (64.6)	510 (63.9)	603 (63.1)	703 (59.4)	818 (66.3)	<0.001
BES	233 (3.8)	75 (21.6)	29 (6.9)	51 (8.7)	38 (5.5)	11 (1.4)	9 (0.9)	18 (1.5)	2 (0.2)	<0.001
CMV, mean (SD)	130.09 (75.66)	158.18 (90.05)	150.87 (86.27)	150.00 (84.39)	146.33 (85.15)	127.19 (72.98)	120.86 (67.19)	115.27 (64.87)	120.48 (68.03)	<0.001
**Biochemical characteristics**										
eGFR, mean (SD)	76.9 (27.3)	70.7 (21.8)	69.3 (24.6)	69.1 (22.0)	75.3 (26.3)	79.5 (28.6)	81.2 (29.9)	78.2 (27.8)	79.8 (27.1)	<0.001
HGB, mean (SD)	123.4 (14.5)	123.4 (14.9)	123.6 (15.3)	125.4 (14.5)	123.9 (14.9)	123.4 (14.2)	123.2 (14.3)	123.1 (14.0)	122.8 (14.7)	0.009
LDL-C, median [IQR]	2.71 [2.12, 3.38]	2.64 [2.06, 3.18]	2.34 [1.86, 2.85]	2.81 [2.23, 3.54]	2.79 [2.21, 3.41]	2.61 [2.09, 3.27]	2.71 [2.14, 3.44]	2.81 [2.16, 3.51]	2.75 [2.15, 3.46]	<0.001
HDLC, median [IQR]	1.07 [0.90, 1.26]	1.18 [1.02, 1.38]	1.16 [0.99, 1.33]	1.11 [0.94, 1.31]	1.09 [0.90, 1.28]	0.99 [0.83, 1.16]	1.03 [0.87, 1.20]	1.08 [0.91, 1.26]	1.07 [0.92, 1.26]	<0.001
HbA1c, mean (SD)	6.70 (1.43)	6.78 (1.50)	6.68 (1.27)	6.71 (1.42)	6.68 (1.35)	6.70 (1.37)	6.90 (1.60)	6.65 (1.36)	6.63 (1.46)	0.415
LVEF, mean (SD)	61.9 (11.0)	63.5 (11.2)	61.5 (13.1)	64.1 (10.5)	62.7 (10.7)	61.4 (11.6)	61.2 (10.7)	60.8 (10.8)	61.9 (10.2)	<0.001
**Medication**, ***n*** **(%)**										
ACEI/ARB	3,009 (49.7)	190 (57.6)	236 (59.6)	358 (62.3)	366 (54.3)	420 (54.0)	450 (48.3)	483 (41.7)	506 (41.9)	<0.001
β-blocker	4,898 (80.9)	254 (77.0)	329 (83.1)	474 (82.4)	567 (84.1)	637 (81.9)	790 (84.8)	902 (77.8)	945 (78.2)	0.0221
Statins	5,539 (91.5)	284 (86.1)	354 (89.4)	527 (91.7)	629 (93.3)	716 (92.0)	871 (93.5)	1,045 (90.2)	1,113 (92.1)	0.0502
Aspirin	5,241 (86.6)	279 (84.5)	350 (88.4)	512 (89.0)	601 (89.2)	686 (88.2)	804 (86.3)	970 (83.7)	1,039 (85.9)	0.0175
Clopidogrel	4,732 (78.2)	271 (82.1)	326 (82.3)	483 (84.0)	546 (81.0)	610 (78.4)	727 (78.0)	833 (71.9)	936 (77.4)	<0.001
CCB	1,679 (27.7)	84 (25.5)	127 (32.1)	182 (31.7)	189 (28.0)	206 (26.5)	205 (22.0)	352 (30.4)	334 (27.6)	0.379

*ACEI/ARB, angiotensin-converting enzyme inhibitor/angiotensin receptor blocker. CCB, calcium channel blocker; CHF, congestive heart failure; CKD, chronic kidney disease; DM, diabetes mellitus; eGFR, estimated glomerular filtration rate; HbA1c, hemoglobin A1c; LDL-C, low-density lipoprotein cholesterol; LVEF, left ventricular ejection fraction; PCI, percutaneous coronary intervention*.

There was no significant difference in the 5-year mortality rate between men and women during the first period (2007–2010), but it was significantly higher in men than in women during the second period (2011–2014). During 2007–2010, the incidence of CHF and the use of β-blocker were significantly higher in men than women while during 2011–2014, there are no differences between men and women. The differences in other characteristics between men and women in the two periods (2007–2010 and 2011–2014) are similar. More details are shown in Supplementary Table 1.

### Trends of the Proportion of Each Age Subgroup Among All Patients With CAD

**Figure 2:**
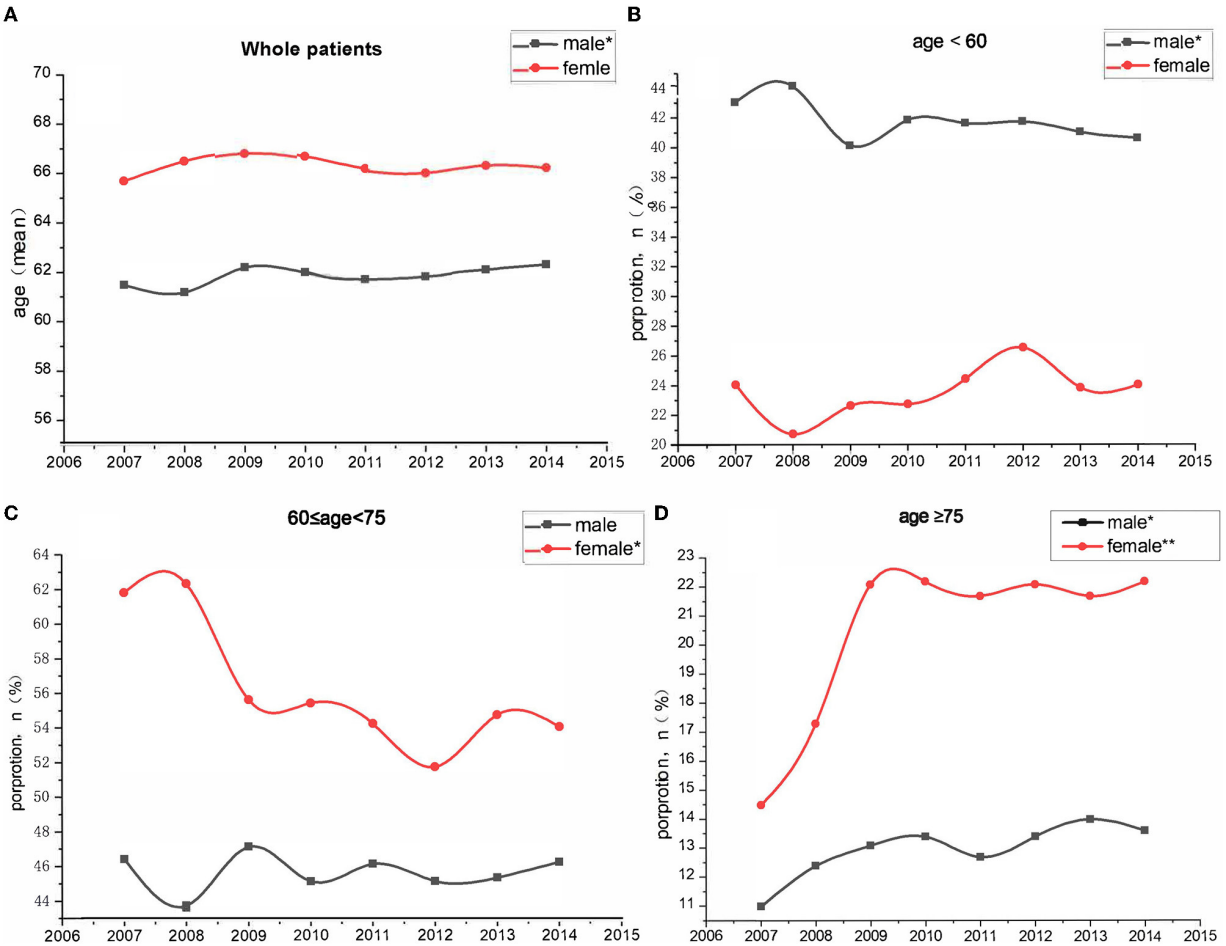
Trends in proportion of different age group among patients with coronary artery disease between 2007 and 2014. **(A)** Whole patients; **(B)** age < 60-year group; **(C)** 60 ≤ age < 5-year group; **(D)** age ≥ 75-year group. ^*^P < 0.05; ^**^P < 0.001.

From 2007 to 2014, the mean age gradually increased from 61.4 to 62.2 among the women whereas the mean age did not increase among the women. The proportion of the age < 60-year group significantly declined from 42.9 to 40.5 % among the male group whereas there was no significant change among female group. The proportion of the 60 ≤ age < 75-year group significantly declined from 61.7 to 53.9% among the female group while there was no significant change among male group. Similarly, the proportion of the 60 ≤ age <75-year group significantly declined from 61.7 to 53.9% among the female group while there was no significant change among male group. However, the proportion of the age ≥ 75-year group significantly increased from 10.9 to 13.5% among the male group and increased from 14.4 to 22.1% among female group (Figure 2).

### Trends of 1-Year Mortality Among All Patients With CAD and Within Each Age Subgroup

**Figure 3:**
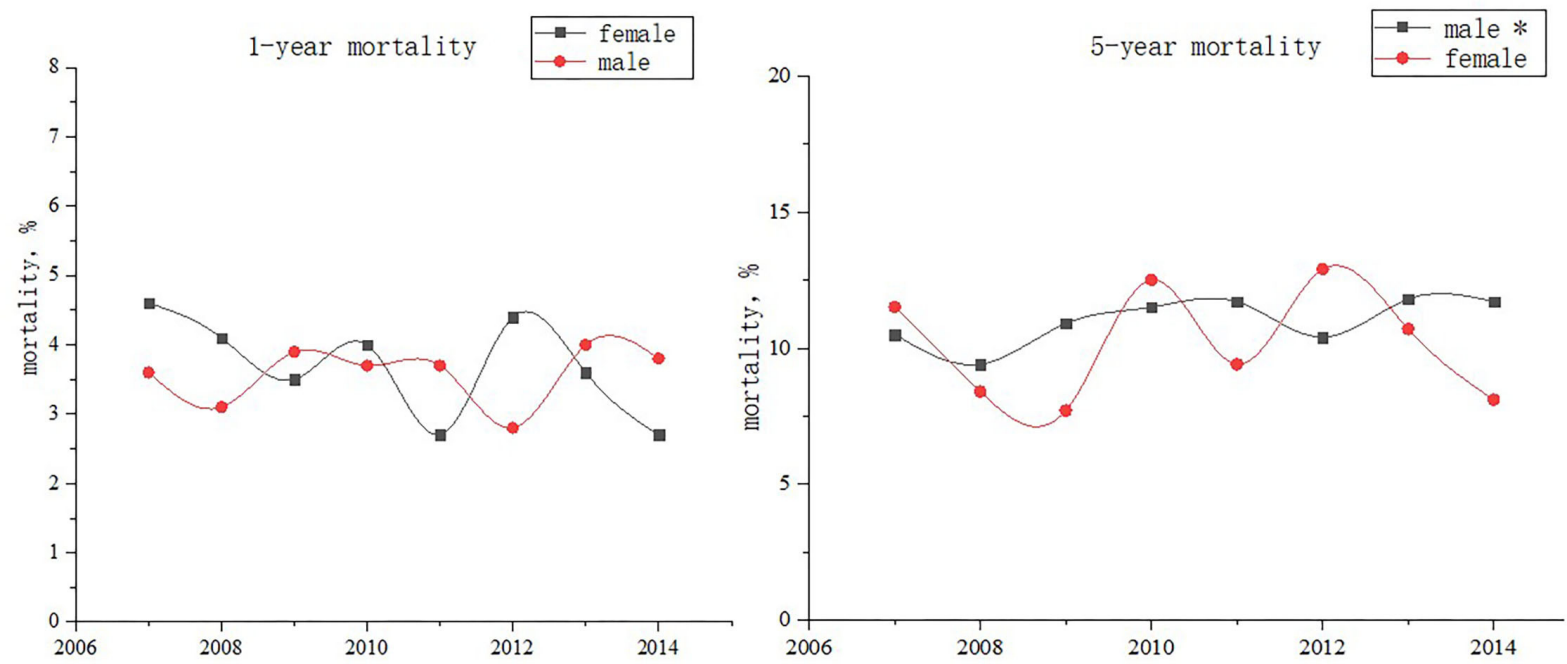
1-year and 5-year mortality among patients with coronary artery disease stratified by sex between 2007 and 2014. ^*^p for trend < 0.001.

From 2007 to 2014, 1-year age-standardized mortality did not significantly change from 3.5 to 3.8% in men (*p* for trend = 0.49) and from 4.3 to 2.5% in women (*p* for trend = 0.19). In women, the mortality decreased from 3.6 to 1.0% among age < 60-year group (*p* for trend =0.04) whereas the mortality did not significantly change in both age ≥75-year group (*p* for trend = 0.55) and 60 ≤ age < 75-year group (*p* for trend = 0.74). In addition, no significant difference was found in either age group among men. The multivariable-adjusted hazard ratios (95% CI), which compare women with men, were from 1.02 (0.39–2.67) to 0.66 (0.39–0.12) for 1-year all-cause mortality (Figure 3 and Table 4).

**Table 4:** Trend in 1-year Mortality (2007–2014).

**Years**	**Female/male, death**	aHR^*^	**95%Cl**	***P* for trend**
2007	15/45	1.02	0.39–2.67	0.14
2008	16/43	2.01	0.89–4.57	
2009	21/74	0.84	0.43-1.65	
2010	29/77	1.04	0.51–2.12	
2011	22/85	0.89	0.40–1.98	
2012	43/75	1.09	0.65–1.83	
2013	42/132	0.76	0.47–1.21	
2014	31/130	0.66	0.39–1.12	

* *Adjusted for age,insurance type, hypertension, CKD, acute myocardial infarction, congestive heart failure, stroke, diabetes mellitus, percutaneous coronary intervention, AF COPD, cancer,and anemia*.

### Trends of 5-Year Mortality Among All Patients With CAD and Within Each Age Subgroup

**Figure 4:**
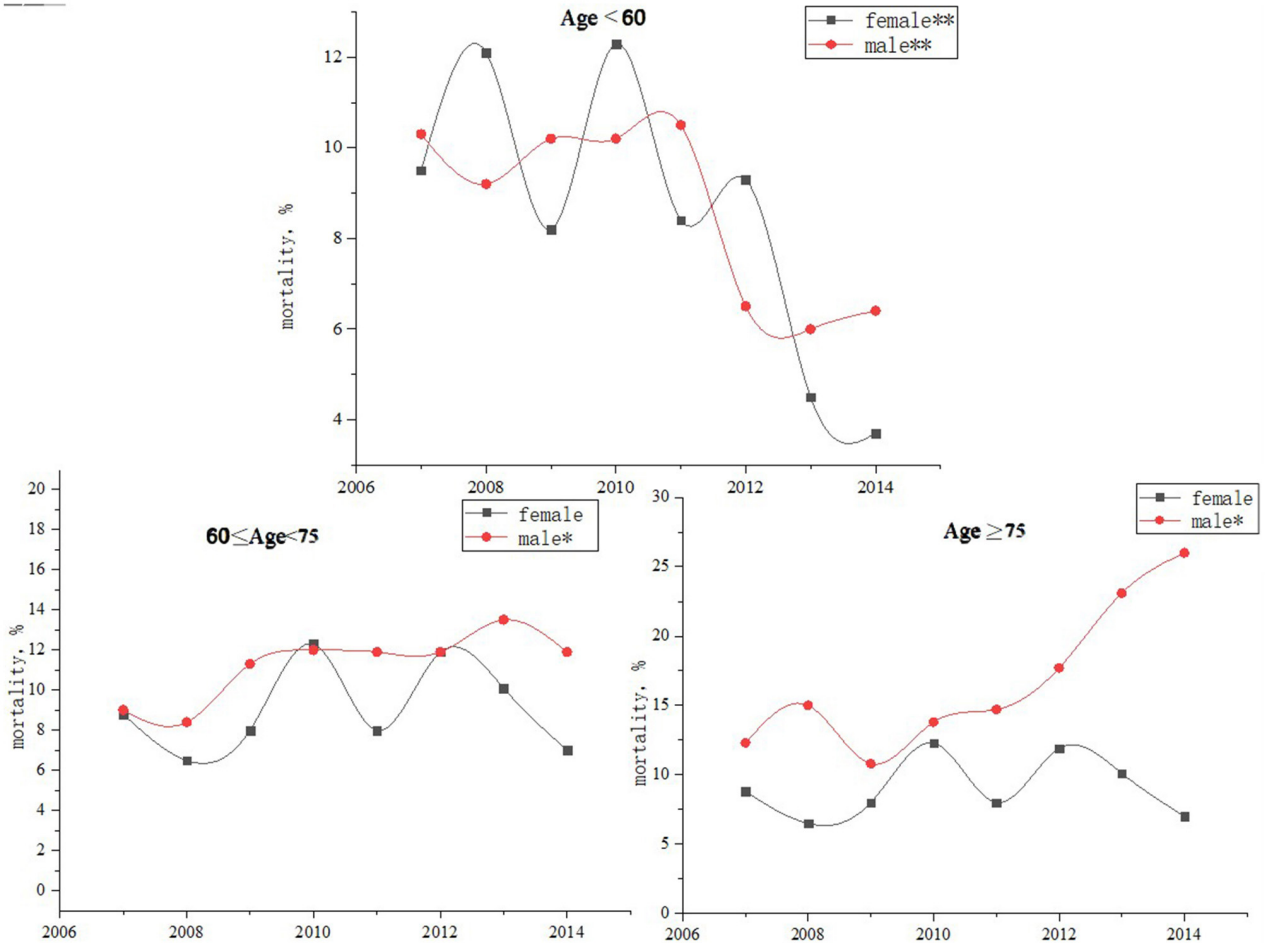
Trends in the 5-year mortality of different age groups among patients with coronary artery disease stratified by sex, from 2007 to 2014. *p for tend < 0.01 **p for trend < 0.001.

From 2007 to 2014, 5-year age-standardized mortality increased from 10.0 to 11.7% in men (*p* for trend < 0.001) and decreased from 11.5 to 8.1% in women (*p* for trend = 0.99). In men, the mortality decreased from 10.4 to 6.6% among age < 60-year group (*p* for trend =0.03) whereas the mortality increased in both age ≥ 75-year group (*p* for trend = 0.005) and 60 ≤ age < 75-year group (*p* for trend = 0.02). The multivariable-adjusted hazard ratios (95% CI), which compare women with men, were from1.23 (0.64–2.36) to 0.59 (0.44–0.79) for 5-year all-cause mortality (*p* for trend = 0.04) (Figures 3, 4 and Table 5).

**Table 5:** Trend in 5-year mortality (2007–2014).

**Years**	**Female/male, death**	aHR^*^	**95%Cl**	***P* for trend**
2007	40/121	1.23	0.64–2.36	0.04
2008	35/383	0.91	0.54–1.53	
2009	45/203	0.72	0.47–1.11	
2010	86/241	1.04	0.70–1.56	
2011	75/268	0.78	0.52–1.18	
2012	123/280	0.90	0.67–1.22	
2013	127/385	0.78	0.59–1.02	
2014	100/395	0.59	0.44–0.79	

* *Adjusted for demographic characteristics (age and insurance type), diagnosis (hypertension, CKDs, acute myocardial infarction, congestive heart failure, stroke, diabetes mellitus, percutaneous coronary intervention, AF COPD, cancer, and anemia)*.

## Discussion

Our study found that the mortality risk among men and women was similar in the 1-year prognosis of CAD, and there was no significant downward trend. In the 5-year long-term prognosis of CAD, the mortality risk among men continued to rise, while women had reached the peak, which means that the mortality risk in men and women is still expanding.

Global Burden of Disease study indicated that CAD remains the leading cause of morbidity and mortality (1). Understanding sex difference temporal trends of mortality in patients with CAD remains a research gap in the largest developing country worldwide. The previous studies indicate that CAD mortality is higher among men than women. Encouragingly, among both US men and women, the age-adjusted mortality rates from CAD have decreased steadily from 1980 through 2002 and the gap is narrowing (14). Similar results have proved that over the past 4 decades, the sex difference of CAD in European and American countries has been narrowed in keeping with a previous WHO report (8, 9, 14).

The association between gender and mortality among patients with cardiovascular disease has been a major topic of study in the past several decades. Despite the increased attention, this relation is poorly understood, and we found contrasting results in the published data. For example, in the studies by Berger et al. (15) and Singh et al. (16), the mortality rates were similar. However, in the recent report by Khera et al. (17), greater rates of mortality were demonstrated in the women. These different findings could have been related to the different years of selection, follow-up length, and countries. Some of these studies reported higher recurrence or mortality rates after MI in women than those in men, whereas others showed better or similar prognosis after MI among women. A pooled analysis of 35 studies, which involved nearly 60,000 patients with ST-segment elevation MI who were treated with primary percutaneous coronary intervention, found that the higher risk of 1-year mortality in women compared with that of men was explained by the differences in baseline cardiovascular risk factors and their clinical profiles (18). Most studies have yielded conflicting results on sex-related outcomes (19–21).

However, it has not been established whether the sex difference trend of long-term mortality trends similarly exists. In this context, we would assess sex difference temporal trends of mortality in China, the largest developing country with the highest burden of ischemic heart disease (1) which still exist apparent gender inequalities in health and well-being.

First, this may be related to different regions, patients' life, and eating habits, which includes Chinese men's living habits, which are not healthy. The WHO's study of 46 different countries and different genders found that the differences in the risk of coronary heart disease between different genders are mainly related to smoking, obesity, high blood pressure, high TC, and low LDL-C, and the gender difference in coronary heart disease mortality can also be determined by these five risk factors that are closely related. Additionally, a large number of clinical studies have shown that men are the risk factors for coronary heart disease. Second, the recent efforts to raise awareness of the burden of cardiovascular disease in women, which includes several women-specific clinical guidelines (4, 22), may have contributed to relatively larger gains among women than men in the recent years. In addition, the proportion of female patients receiving coronary intervention is lower than that of male patients which may be a marker of patient clinical complexity. In addition, the proportion of older women is declining, which may be one of the factors that women have a better prognosis.

Our study suggests that poor prognosis of CAD is still a major public health problem in China and men are still high-risk groups. Furthermore, in our study, women were less likely to receive PCI for CAD as compared to men. This result may raise concerns about reperfusion during hospitalization, the quality of long-term care, and allocation of resources after discharge not only for China but also for other developing countries with a rapidly growing cardiovascular disease burden and irregular long-term treatment. Furthermore, these data highlight the need for continued interventions normatively to ensure that men and women receive guideline-recommended treatment especially the standard treatment after discharge to lower the risk for CAD.

Our study also has some limitations. First, this was a retrospective cohort study that was conducted in a single center. Due to the limitations of research, we cannot collect enough confounder factors, such as economic level, behavioral habits, and more, which limited us to analyze the underlying mechanism. However, our investigation focuses on the most reliable data available, to evaluate the trends in Asian subgroups who represent approximately 100% of the Chinese population. In addition, our so large study cohort that includes more than 20,000 patients who were from all around the southern cities does reflect the sex difference trends in mortality risk of CAD over the decades among Chinese women and men. Second, these data may not reflect the clinical progression of CAD, which especially lacks the specific information on the cause of death. Nonetheless, the large sample size provides a valuable frame to assess the attributable risk of all-cause mortality.

Our study indicated that in the 5-year long-term prognosis of CAD, the mortality risk among men continued to rise, while women had reached the peak, which means that the mortality risk continues to be higher among men than women. The challenge to further improve treatment of both male and female patients with CAD is to strengthen multispecialty integration and long-term management.

## Data Availability Statement

The original contributions presented in the study are included in the article/Supplementary Materials, further inquiries can be directed to the corresponding author/s.

## Ethics Statement

The studies involving human participants were reviewed and approved by Guangdong Provincial People's Hospital Ethics Committee. The Ethics Committee waived the requirement of written informed consent for participation.

## Author Contributions

YL, JL, JC, HY, and C-QO designed the study. QL, ZYan, BW, and YH collected, reviewed clinical, and laboratory data. SC, HW, NR, and TT analyzed data. SC, ZYang, C-QO, and KB performed the statistical analysis. QL, HH, WL, HW, NR, and KB drafting or revising of the manuscript. YL, JL, JC, and HY reviewed, interpreted, and checked clinical data. All authors contributed to the article and approved the submitted version.

## Funding

This research was funded and supported by the National Key Research and Development Program of China, Grant (2016YFC1301202); multi-center study on key techniques for prevention, diagnosis, and treatment of high risk CAD (DFJH2020026).

## Conflict of Interest

The authors declare that the research was conducted in the absence of any commercial or financial relationships that could be construed as a potential conflict of interest.

## Publisher's Note

All claims expressed in this article are solely those of the authors and do not necessarily represent those of their affiliated organizations, or those of the publisher, the editors and the reviewers. Any product that may be evaluated in this article, or claim that may be made by its manufacturer, is not guaranteed or endorsed by the publisher.

## Supplementary Material

Supplementary Figure 1 The flow of participants through the trial.

Supplementary Table 1 Clinical characteristics of patients with CAD in different time periods.

## References

[1] Dai H Much AA Maor E Asher E Younis A Xu Y. Global, regional, and national burden of ischemic heart disease and its attributable risk factors, 1990–2017: results from the global burden of disease study 2017. Eur Heart J Qual Care Clin Outcomes. (2020) 8:50–60. 10.1093/ehjqcco/qcaa076 33017008 PMC8728029

[2] Paul TK Sivanesan K Schulman-Marcus J. Sex differences in nonobstructive coronary artery disease: recent insights and substantial knowledge gaps. Trends Cardiovasc Med. (2017) 27:173–9. 10.1016/j.tcm.2016.08.002 27617797

[3] Chambers TA Bagai A Ivascu N. Current trends in coronary artery disease in women. Curr Opin Anaesthesiol. (2007) 20:75–82. 10.1097/ACO.0b013e3280146455 17211172

[4] Mosca L Barrett-Connor E Wenger NK. Sex/gender differences in cardiovascular disease prevention: what a difference a decade makes. Circulation. (2011) 124:2145–54. 10.1161/CIRCULATIONAHA.110.968792 22064958 PMC3362050

[5] Pepine CJ Ferdinand KC Shaw LJ Light-McGroary KA Shah RU Gulati M. Emergence of non-obstructive coronary artery disease: a woman's problem and need for change in definition on angiography. J Am Coll Cardiol. (2015) 66:1918–33. 10.1016/j.jacc.2015.08.876 26493665 PMC4618799

[6] Wilmot KA O'Flaherty M Capewell S Ford ES Vaccarino V. Coronary heart disease mortality declines in the united states from 1979 through 2011: evidence for stagnation in young adults, especially women. Circulation. (2015) 1 32:997–1002. 10.1161/CIRCULATIONAHA.115.015293 26302759 PMC4828724

[7] Szummer K Wallentin L Lindhagen L Alfredsson J Erlinge D Held C. Improved outcomes in patients with ST-elevation myocardial infarction during the last 20 years are related to implementation of evidence-based treatments: experiences from the SWEDEHEART registry 1995-2014. Eur Heart J. (2017) 38:3056–65. 10.1093/eurheartj/ehx515 29020314 PMC5837507

[8] Mathers CD Sadana R Salomon JA Murray CJ Lopez AD. Healthy life expectancy in 191 countries, 1999. Lancet (London, England). (2001) 357:1685–91. 10.1016/S0140-6736(00)04824-8 11425392

[9] Hartley A Marshall DC Salciccioli JD Sikkel MB Maruthappu M Shalhoub J. Trends in mortality from ischemic heart disease and cerebrovascular disease in europe: 1980 to 2009. Circulation. (2016) 133:1916–26. 10.1161/CIRCULATIONAHA.115.018931 27006480

[10] Kushner FG Hand M Smith SC King SB Anderson JL Antman EM. 2009 focused updates: ACC/AHA guidelines for the management of patients with ST-elevation myocardial infarction (updating the 2004 guideline and 2007 focused update) and ACC/AHA/SCAI guidelines on percutaneous coronary intervention (updating the 2005 guideline and 2007 focused update) a report of the american college of cardiology foundation/american heart association task force on practice guidelines. J Am Coll Cardiol 2009, 54:2205–41. 10.1016/j.jacc.2009.10.015 19942100

[11] Jneid H Anderson JL Wright RS Adams CD Bridges CR Casey DE. 2012 ACCF/AHA focused update of the guideline for the management of patients with unstable angina/non-ST-elevation myocardial infarction (updating the 2007 guideline and replacing the 2011 focused update): a report of the american college of cardiology foundation/american heart association task force on practice guidelines. J Am Coll Cardiol. (2012) 60:645–81. 10.1161/CIR.0b013e318256f1e0 22809746

[12] Levine GN Bates ER Blankenship JC Bailey SR Bittl JA Cercek B. 2015 ACC/AHA/SCAI focused update on primary percutaneous coronary intervention for patients with ST-elevation myocardial infarction: an update of the 2011 ACCF/AHA/SCAI guideline for percutaneous coronary intervention and the 2013 ACCF/AHA guideline for the management of ST-elevation myocardial infarction. J Am Coll Cardiol. (2016) 67:1235–50. 10.1002/ccd.26325 26498666

[13] Aguiar-Souto P Ferrante G Del Furia F Barlis P Khurana R Di Mario C. Frequency and predictors of contrast-induced nephropathy after angioplasty for chronic total occlusions. Int J Cardiol. (2010) 139:68–74. 10.1016/j.ijcard.2008.10.006 19056138

[14] Ford ES Capewell S. Coronary heart disease mortality among young adults in the U.S. from 1980 through 2002: concealed leveling of mortality rates. J Am Coll Cardiol. (2007) 50:2128–32. 10.1016/j.jacc.2007.05.056 18036449

[15] Berger JS Sanborn TA Sherman W Brown DL. Influence of sex on in-hospital outcomes and long-term survival after contemporary percutaneous coronary intervention. Am Heart J. (2006) 151:1026–31. 10.1016/j.ahj.2004.05.062 16644329

[16] Singh M Rihal CS Gersh BJ Roger VL Bell MR Lennon RJ. Mortality differences between men and women after percutaneous coronary interventions. a 25-year, single-center experience. J Am Coll Cardiol. (2008) 51:2313–20. 10.1016/j.jacc.2008.01.066 18549915 PMC2733245

[17] Khera S Kolte D Gupta T Subramanian KS Khanna N Aronow WS. Temporal trends and sex differences in revascularization and outcomes of st-segment elevation myocardial infarction in younger adults in the United States. J Am Coll Cardiol. (2015) 66:1961–72. 10.1016/j.jacc.2015.08.865 26515998

[18] Pancholy SB Shantha GP Patel T Cheskin LJ. Sex differences in short-term and long-term all-cause mortality among patients with ST-segment elevation myocardial infarction treated by primary percutaneous intervention: a meta-analysis. JAMA Intern Med. (2014) 174:1822–30. 10.1001/jamainternmed.2014.4762 25265319

[19] Batchelor W Kandzari DE Davis S Tami L Wang JC Othman I. Outcomes in women and minorities compared with white men 1 year after everolimus-eluting stent implantation: insights and results from the PLATINUM diversity and PROMUS element plus post-approval study pooled analysis. JAMA cardiology. (2017) 2:1303–13. 10.1001/jamacardio.2017.3802 29049508 PMC5814993

[20] Heer T Hochadel M Schmidt K Mehilli J Zahn R Kuck K. Sex differences in percutaneous coronary intervention-insights from the coronary angiography and PCI registry of the german society of cardiology. J Am Heart Assoc. (2017) 6:e004972 10.1161/JAHA.116.004972 28320749 PMC5524024

[21] Kunadian V Qiu W Lagerqvist B Johnston N Sinclair H Tan Y. Gender differences in outcomes and predictors of all-cause mortality after percutaneous coronary intervention (data from United Kingdom and Sweden). Am J Cardiol. (2017) 119:210–6. 10.1016/j.amjcard.2016.09.052 27816119

[22] Mehta LS Beckie TM DeVon HA Grines CL Krumholz HM Johnson MN. Acute myocardial infarction in women: a scientific statement from the american heart association. Circulation. (2016) 133:916–47. 10.1161/CIR.0000000000000351 26811316

