# Supplementary figures and images for: Sex Difference Trend in 5-Year Mortality Among Patients With Coronary Artery Disease: A 24,432 Chinese Cohort Study From 2007 to 2014
Source: Front Cardiovasc Med. 2022 Apr 12;9:774365. doi: 10.3389/fcvm.2022.774365 (PMC9039363; doi:10.3389/fcvm.2022.774365)

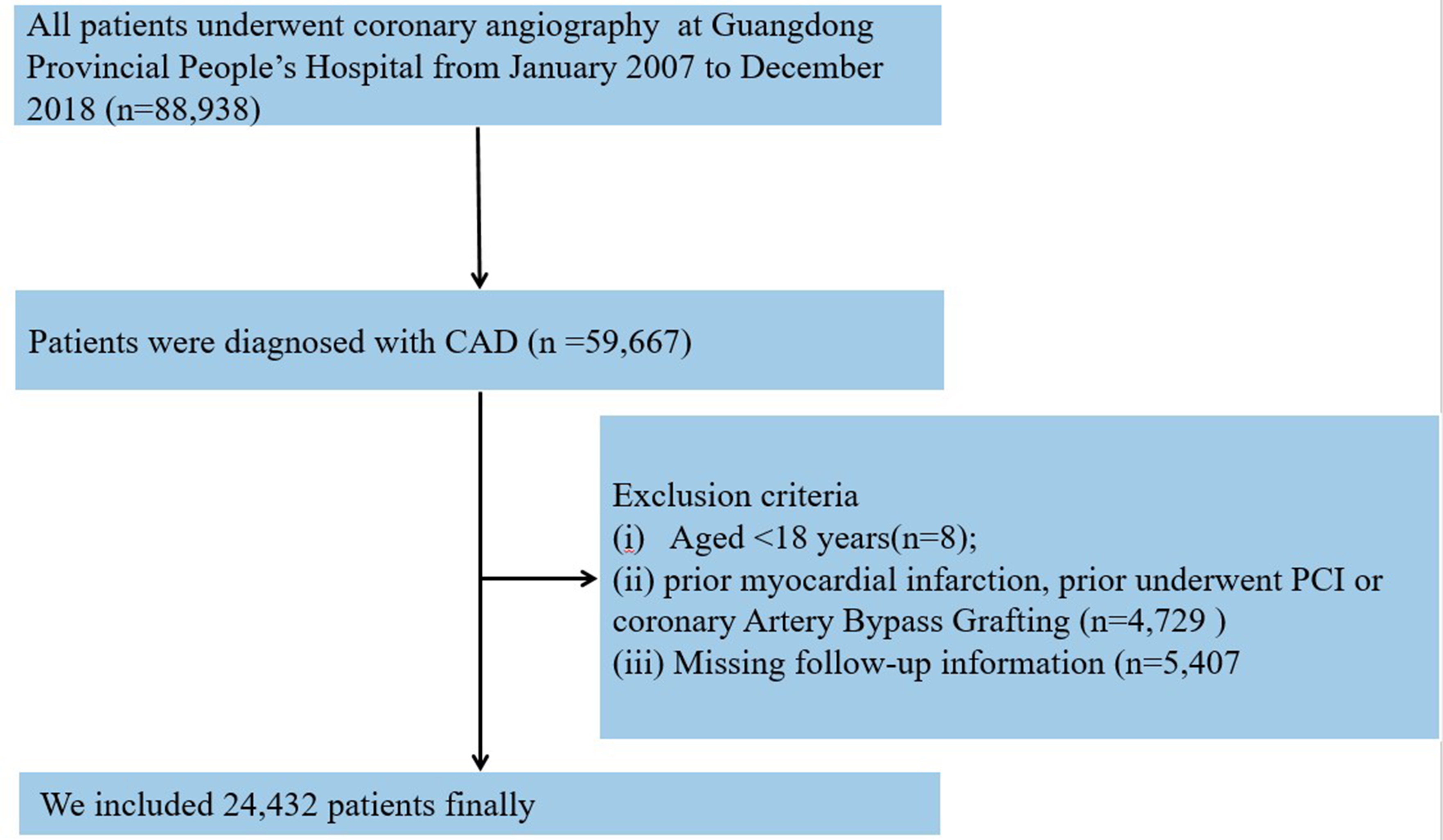

Supplement: Supplementary Figure 1 — The flow of participants through the trial. [file Image_1.JPEG]
