# Supplementary material for: Sex Difference Trend in 5-Year Mortality Among Patients With Coronary Artery Disease: A 24,432 Chinese Cohort Study From 2007 to 2014
Source: Front Cardiovasc Med. 2022 Apr 12;9:774365. doi: 10.3389/fcvm.2022.774365 (PMC9039363; doi:10.3389/fcvm.2022.774365)
Supplement: Supplementary Table 1 — Clinical characteristics of patients with CAD in different time periods. [file Table_1.docx]

**Supplement Table 1.** Clinical characteristics of CAD patients in different time periods.

| **Characteristic** | 2007-2010 | | | 2011-2014 | | | P Value  2007-2010 | P Value  2011-2014 |
| --- | --- | --- | --- | --- | --- | --- | --- | --- |
|  | Overall  (N=8,643) | Male  (N=6,605) | Female  (N=2,038) | Overall  (N=15,789) | Male  (N=11,619) | Female  (N=4,170) |  |  |
| **Demographics, n (%)** | | | | | | | |  |
| Age, (years), mean (SD) | 62.81 (10.83) | 61.68 (10.98) | 66.46 (9.44) | 63.05 (10.66) | 61.93 (10.80) | 66.15 (9.60) | <0.001 | <0.001 |
| <60 | 3225 (37.3) | 2769 (41.9) | 456 (22.4) | 5795 (36.7) | 4773 (41.1) | 1022 (24.5) | <0.001 | <0.001 |
| 60-75 | 4185 (48.4) | 3006 (45.5) | 1179 (57.9) | 7531 (47.7) | 5294 (45.6) | 2237 (53.6) |  |  |
| ≥75 | 1233 (14.3) | 830 (12.6) | 403 (19.8) | 2463 (15.6) | 1552 (13.4) | 911 (21.8) |  |  |
| gender (male, %) | 6605 (76.4) | 6605 (100.0) | 0 (0.0) | 11619 (73.6) | 11619 (100.0) | 0 (0.0) | <0.001 | <0.001 |
| **Insurance Type, n (%)** | | | | | | | |  |
| Self-paying | 3327 (38.5) | 2640 (40.0) | 687 (33.7) | 9431 (60.0) | 6972 (60.2) | 2459 (59.2) | <0.001 | <0.001 |
| Urban insurance | 3737 (43.2) | 2746 (41.6) | 991 (48.6) | 773 (4.9) | 585 (5.1) | 188 (4.5) |  |  |
| Rural insurance | 634 (7.3) | 513 (7.8) | 121 (5.9) | 3413 (21.7) | 2416 (20.9) | 997 (24.0) |  |  |
| other | 945 (10.9) | 706 (10.7) | 239 (11.7) | 15229 (96.5) | 11197 (96.4) | 4032 (96.7) |  |  |
| **Discharge Status, n (%)** | | | | | | | |  |
| Medical advice discharge | 8470 (98.0) | 6489 (98.2) | 1981 (97.2) | 15589 (98.7) | 11472 (98.7) | 4117 (98.7) | 0.006 | <0.001 |
| Automatic withdraw | 103 (1.2) | 74 (1.1) | 29 (1.4) | 113 (0.7) | 80 (0.7) | 33 (0.8) |  |  |
| In-hospital death | 55 (0.6) | 34 (0.5) | 21 (1.0) | 36 (0.2) | 24 (0.2) | 12 (0.3) |  |  |
| **Discharge Diagnosis, n (%)** | | | | | | | |  |
| AMI | 2110 (24.4) | 1788 (27.1) | 322 (15.8) | 3234 (20.5) | 2658 (22.9) | 576 (13.8) | <0.001 | <0.001 |
| HT | 4629 (53.6) | 3283 (49.7) | 1346 (66.0) | 8987 (56.9) | 6187 (53.2) | 2800 (67.1) | <0.001 | <0.001 |
| DM | 2108 (24.4) | 1477 (22.4) | 631 (31.0) | 4184 (26.5) | 2833 (24.4) | 1351 (32.4) | <0.001 | <0.001 |
| AF | 238 (2.8) | 162 (2.5) | 76 (3.7) | 463 (2.9) | 312 (2.7) | 151 (3.6) | <0.001 | 0.003 |
| CHF | 410 (9.3) | 313 (9.6) | 97 (8.4) | 1362 (19.6) | 1004 (20.2) | 358 (18.1) | <0.001 | 0.053 |
| ICD_STROKE | 349 (4.0) | 262 (4.0) | 87 (4.3) | 813 (5.1) | 593 (5.1) | 220 (5.3) | 0.588 | 0.696 |
| ICD_CANCER | 108 (1.2) | 81 (1.2) | 27 (1.3) | 160 (1.0) | 103 (0.9) | 57 (1.4) | 0.814 | 0.01 |
| CKD | 2010 (25.8) | 1433 (24.3) | 577 (30.9) | 3169 (20.7) | 2218 (19.7) | 951 (23.5) | <0.001 | <0.001 |
| **Procedure, n (%)** | | | | | | | |  |
| PCI | 6424 (74.3) | 5010 (75.9) | 1414 (69.4) | 11582 (73.4) | 8882 (76.4) | 2700 (64.7) | <0.001 | <0.001 |
| DES | 5636 (65.2) | 4402 (66.6) | 1234 (60.5) | 11221 (71.1) | 8587 (73.9) | 2634 (63.2) | <0.001 | <0.001 |
| BES | 873 (10.1) | 680 (10.3) | 193 (9.5) | 241 (1.5) | 201 (1.7) | 40 (1.0) | 0.299 | <0.001 |
| **Biochemical characteristics** | | | | | | | |  |
| CMV, mean (SD) | 160.56 (91.09) | 163.76 (92.38) | 150.28 (86.03) | 135.89 (75.97) | 141.48 (77.87) | 120.39 (68.05) | <0.001 | <0.001 |
| eGFR, mean (SD) | 73.94 (22.68) | 74.72 (22.07) | 71.52 (24.32) | 80.56 (25.92) | 80.90 (25.04) | 79.59 (28.25) | <0.001 | 0.012 |
| WBC, mean (SD) | 8.29 (3.13) | 8.44 (3.17) | 7.82 (2.92) | 8.07 (2.82) | 8.26 (2.84) | 7.56 (2.71) | <0.001 | <0.001 |
| HGB, mean (SD) | 133.59 (16.81) | 136.53 (16.28) | 124.20 (14.90) | 132.80 (16.69) | 136.29 (16.11) | 123.09 (14.28) | <0.001 | <0.001 |
| LDL-C, median [IQR] | 2.60 [2.06, 3.21] | 2.58 [2.05, 3.19] | 2.66 [2.09, 3.29] | 2.69 [2.10, 3.37] | 2.67 [2.09, 3.35] | 2.73 [2.14, 3.43] | 0.003 | <0.001 |
| HDLC, median [IQR] | 1.02 [0.84, 1.21] | 0.98 [0.82, 1.17] | 1.13 [0.95, 1.32] | 0.95 [0.81, 1.12] | 0.92 [0.79, 1.07] | 1.05 [0.89, 1.23] | <0.001 | <0.001 |
| HbA1c, mean (SD) | 6.54 (1.32) | 6.49 (1.30) | 6.70 (1.37) | 6.56 (1.42) | 6.51 (1.40) | 6.71 (1.45) | <0.001 | <0.001 |
| LVEF, mean (SD) | 60.13 (12.13) | 59.25 (12.25) | 62.97 (11.29) | 59.42 (11.66) | 58.75 (11.90) | 61.32 (10.76) | <0.001 | <0.001 |
| **Biochemical characteristics** | | | | | | | |  |
| ACEI/ARB | 5394 (64.2) | 4244 (66.0) | 1150 (58.2) | 7800 (50.4) | 5941 (52.2) | 1859 (45.6) | <0.001 | 0.001 |
| β-blocker | 6999 (83.3) | 5375 (83.6) | 1624 (82.2) | 12682 (82.0) | 9408 (82.6) | 3274 (80.3) | 0.161 | <0.001 |
| Statins | 7771 (92.5) | 5977 (93.0) | 1794 (90.8) | 14633 (94.6) | 10888 (95.6) | 3745 (91.8) | <0.001 | <0.001 |
| Aspirin | 7642 (90.9) | 5900 (91.8) | 1742 (88.2) | 13944 (90.1) | 10445 (91.7) | 3499 (85.8) | 0.002 | <0.001 |
| Clopidogrel | 7329 (87.2) | 5703 (88.7) | 1626 (82.3) | 13051 (84.4) | 9945 (87.3) | 3106 (76.2) | <0.001 | <0.001 |
| CCB | 1959 (23.3) | 1377 (21.4) | 582 (29.5) | 3246 (21.0) | 2149 (18.9) | 1097 (26.9) | <0.001 | <0.001 |
| **Follow up death, n (%)** | | | | | | | |  |
| 1-year follow up death | 317 (3.7) | 236 (3.6) | 81 (4.0) | 560 (3.5) | 422 (3.6) | 138 (3.3) | 0.438 | 0.359 |
| 5-years Follow up death | 905 (10.5) | 699 (10.6) | 206 (10.1) | 1753 (11.1) | 1328 (11.4) | 425 (10.2) | 0.568 | 0.031 |

**Abbreviation**: ACEI/ARB, angiotensin-converting enzyme inhibitor/angiotensin receptor blocker. CCB, calcium channel blocker; CHF, congestive heart failure; CKD, chronic kidney disease; DM, diabetes mellitus; eGFR, estimated glomerular filtration rate; HbA1c, hemoglobin A1c; LDL-C, low-density lipoprotein cholesterol; LVEF, left ventricular ejection fraction; PCI, percutaneous coronary intervention.
